# Interaction between Dietary Fat Intake and Metabolic Genetic Risk Score on 25-Hydroxyvitamin D Concentrations in a Turkish Adult Population

**DOI:** 10.3390/nu14020382

**Published:** 2022-01-17

**Authors:** Kubra Isgin-Atici, Buthaina E. Alathari, Busra Turan-Demirci, Suleyman Nahit Sendur, Incilay Lay, Basma Ellahi, Mehmet Alikasifoglu, Tomris Erbas, Zehra Buyuktuncer, Karani Santhanakrishnan Vimaleswaran

**Affiliations:** 1 Department of Nutrition and Dietetics, Faculty of Health Sciences, Hacettepe University, Ankara 06230, Turkey; k.isginatici@gmail.com (K.I.-A.); busraturan@hacettepe.edu.tr (B.T.-D.); 2 Department of Nutrition and Dietetics, Faculty of Health Sciences, Amasya University, Amasya 05000, Turkey; 3 Hugh Sinclair Unit of Human Nutrition, Department of Food and Nutritional Sciences, University of Reading, Reading RG6 6DZ, UK; b.e.a.a.alathari@pgr.reading.ac.uk; 4 Department of Food Science and Nutrition, Faculty of Health Sciences, The Public Authority for Applied Education and Training, AlFaiha 72853, Kuwait; 5 Department of Endocrinology and Metabolism, School of Medicine, Hacettepe University, Ankara 06230, Turkey; snahitsendur@hotmail.com (S.N.S.); erbast@hacettepe.edu.tr (T.E.); 6 Department of Medical Biochemistry, Faculty of Medicine, Hacettepe University, Ankara 06230, Turkey; lincilay@gmail.com; 7 Clinical Pathology Laboratory, Hacettepe University Hospitals, Ankara 06230, Turkey; 8 Faculty of Health and Social Care, University of Chester, Chester CH1 4DS, UK; b.ellahi@chester.ac.uk; 9 Department of Medical Genetics, School of Medicine, Hacettepe University, Ankara 06230, Turkey; kasif@hacettepe.edu.tr; 10 Genetics Diagnostic Centre, DAMAGEN, Ankara 06230, Turkey; 11 Institute for Food, Nutrition, and Health, University of Reading, Reading RG6 6AH, UK

**Keywords:** vitamin D, *TCF7L2*, *MC4R*, genetic risk score, fat intake, metabolic traits

## Abstract

Previous studies have pointed out a link between vitamin D status and metabolic traits, however, consistent evidence has not been provided yet. This cross-sectional study has used a nutrigenetic approach to investigate the interaction between metabolic-genetic risk score (GRS) and dietary intake on serum 25-hydroxyvitamin D [25(OH)D] concentrations in 396 unrelated Turkish adults, aged 24–50 years. Serum 25(OH)D concentration was significantly lower in those with a metabolic-GRS ≥ 1 risk allele than those with a metabolic-GRS < 1 risk allele (*p* = 0.020). A significant interaction between metabolic-GRS and dietary fat intake (energy%) on serum 25(OH)D levels was identified (*P*_interaction_ = 0.040). Participants carrying a metabolic-GRS ≥ 1 risk allele and consuming a high fat diet (≥38% of energy = 122.3 ± 52.51 g/day) had significantly lower serum 25(OH)D concentration (*p* = 0.006) in comparison to those consuming a low-fat diet (<38% of energy = 82.5 ± 37.36 g/d). In conclusion, our study suggests a novel interaction between metabolic-GRS and dietary fat intake on serum 25(OH)D level, which emphasises that following the current dietary fat intake recommendation (<35% total fat) could be important in reducing the prevalence of vitamin D deficiency in this Turkish population. Nevertheless, further larger studies are needed to verify this interaction, before implementing personalized dietary recommendations for the maintenance of optimal vitamin D status.

## 1. Introduction

Nearly one billion people suffer from vitamin D deficiency (VDD) globally [1]. The prevalence of VDD among adults has been reported as ~40% in Europe [2] and 44–96% in Asia, the Middle East, North Africa, and 30–90% in West Asia [3,4,5,6,7]. Despite having high levels of sun exposure, VDD remains a significant problem in Turkey [8,9]. A meta-analysis of data from 111,582 Turkish participants reported that the prevalence of VDD was 63.5% (58.9–66.6%) in adults, 76% in pregnant women, 39.8% in children, and 86.6% in infants [8]. In addition to the genetic determinants of vitamin D status, personal characteristics such as age, gender, skin colour, race, religious beliefs and clothing style, and lifestyle factors including physical activity level have been suggested as potential factors that can affect the levels of vitamin D in the Turkish population [8,10,11].

As a member of secosteroid hormones, vitamin D plays essential roles in both calcium and phosphorus metabolism, cell proliferation and differentiation, muscle contraction, nerve transmission, and function of the immune system [12]. Due to the immunomodulatory, anti-inflammatory, antifibrotic, and antioxidant roles of vitamin D, its deficiency has associations with several diseases including obesity, diabetes, cardiovascular diseases, bone metabolic disorders, cancers, neuropsychiatric disorders and autoimmune diseases, and more recently with increased risk of SARS-CoV-2 infection [12,13,14]. The link between VDD and the risk of cardiometabolic diseases has been extensively studied [15,16,17], and it has been shown that vitamin D exhibits anti-adipogenic activity in 3T3-L1 preadipocytes [18,19] and has potential roles in inducing the expression of the insulin receptor, in the regulation of insulin secretion, glucose homoeostasis, and inflammation [20,21].

Despite the current evidence for the link between VDD and cardiometabolic diseases, a causal effect has not been established [22]. Furthermore, previous studies investigating this link are inconsistent due to the unmeasured confounding factors [23,24]. A genetic approach may provide a better understanding to the potential association between VDD and metabolic diseases by eliminating any unclear confounding factors [25]. The heritability of circulating vitamin D levels has been reported between 20–85%, and a number of genetic variants in genes for vitamin D pathways have been associated with metabolic diseases [12,26]. Furthermore, several genetic variants associated with cardiometabolic health have also been linked to one’s vitamin D level status. Melanocortin 4 Receptor (*MC4R*) and Transcription Factor 7-Like 2 (*TCF7L2*) genes are commonly studied candidate genes for obesity and diabetes [25,27,28,29,30,31,32,33,34,35,36,37,38,39], and the interactions of *MC4R* and *TCF7L2* genotypes with dietary intakes on obesity [35,36] and diabetes related traits [25,30,37] have been investigated in multiple ethnic groups. However, to our knowledge, the potential effects of the interaction between metabolic-genetic risk score (GRS) and dietary intake on vitamin D status have not been investigated in a Turkish population. Hence, in the present study, we have explored the association of the metabolic-GRS with metabolic traits and vitamin D status and explored the interaction between metabolic-GRS and dietary intake on the vitamin D status of a Turkish population.

## 2. Materials and Methods

### 2.1. Study Population

This cross-sectional study was performed with 396 Turkish adults, aged 24–50 years. The study participants were enrolled following a physical examination by the research endocrinologists at the outpatient clinic of the Department of Endocrinology and Metabolism at the Hacettepe University Hospitals between June and November 2017. Criteria for inclusion required a routine visit to the outpatient clinic, being 24–50 years old, and with a Body Mass Index (BMI) of ≥18.50 kg/m^2^. Those who had diagnosed liver and kidney diseases, mental and psychological disorders, cancers and severe endocrine abnormalities (hypothyroidism, hyperthyroidism, hypopituitarism, etc.), as well as those who were pregnant or breastfeeding, using drugs or dietary supplements that affect body weight, or have a history of bariatric surgery were excluded from the study. Following physical examination, all participants underwent a nutritional assessment and biochemical and genetic analysis. The study was approved by the Non-interventional Clinical Research Ethics Board of Hacettepe University (GO 15/612-11) in compliance with the Declaration of Helsinki, and written informed confirmation was obtained from all the participants. The details of the study, including the procedure for taking blood samples and transport to the laboratory have been previously published [40]. The study was performed as a part of the GeNuIne (Gene-Nutrient Interactions) Collaboration [41,42].

### 2.2. Anthropometrical Measurements

Height and body weight were assessed using standardised methods with a digital scale (Seca 220 Scale). BMI was calculated with the formula: “Body weight (in kilograms) divided by the square of height (in meters)” [43]. Waist circumference (WC) and hip circumference (HC) were measured by standard methods, and the waist-to-hip ratio was calculated by dividing WC (cm) to HC (cm) [44]. Body composition was determined by bioelectrical impedance (Tanita MC- 980 MA). Fat mass index (FMI) was estimated as fat mass (kg)/height squared (m^2^) [45].

### 2.3. Biochemical and Clinical Measures

Fasting lipid profile including triglyceride, total cholesterol, high-density lipoprotein cholesterol (HDL-cholesterol), low-density lipoprotein cholesterol (LDL-cholesterol), and both fasting and postprandial plasma glucose and insulin concentrations were analysed by routine methods at Hacettepe University (Biochemistry Laboratory). Plasma adiponectin and serum 25(OH)D concentrations were analysed in Hacettepe University (Clinical Pathology Laboratory) using ELISA kits (Ebioscience, Austria and Dia Source, Belgium, respectively). According to the Institute of Medicine’s recommendation (IOM) [46], ≥20 ng/mL was considered as an optimal concentration for serum 25(OH)D concentration. Insulin resistance (HOMA-IR) was calculated using the formula: ‘Fasting insulin level (μU /L) x fasting glucose level (nmol/L)/22.5 [47]. Systolic (SBP) and Diastolic (DBP) blood pressure was measured as a part of the physical examination [48].

### 2.4. Dietary Assessment

Two trained research dietitians assessed the dietary intake using the 24-h-dietary recall method. The amount of food items consumed by the participants were confirmed using the food portion size photographic atlas [49], replicas of food items, and household measurement tools. Dietary energy and nutrient intakes were estimated using a dietary analysis computer program (BeBIS, Nutrition Information System, Version 8).

### 2.5. Assessment of Physical Activity Level

A Turkish version of the International Physical Activity Questionnaire (IPAQ) was used to determine the physical activity level of the participants [50]. The physical activity level was categorised into three groups based on Metabolic Equivalent of Task (MET) values suggested by the IPAQ protocol: sedentary (<600 MET/min/w), moderate (600–3000 MET/min/w), and vigorous (>3000 MET/min/w) [51].

### 2.6. Single Nucleotide Polymorphism (SNP) Selection and Genotyping

SNPs, *TCF7L2* rs7903146, and *MC4R* rs571312, were selected because of their associations with metabolic diseases that have been suggested previously in different populations [25,27,28,29,30,31,32,33,34,35,36,37,38,39]. The genomic DNA was isolated from the whole blood in K2EDTA containing tubes by the salting-out method. The details of this method have been described previously [40]. The genotypes of the *TCF7L2* rs7903146 and *MC4R* rs571312 SNPs were in the Hardy–Weinberg equilibrium (*p* = 0.101 and *p* = 0.176, respectively). Genotype distributions and MAFs for the SNPs of *TCF7L2* and *MC4R* are given in Table S1.

### 2.7. Statistical Analysis

The statistical analysis was performed with Statistical Package for the Social Sciences (SPSS) software (version 24). Descriptive data for continuous variables were given as the mean and standard deviation, and groups were compared using the independent sample t test. Allele and genotype frequencies of two SNPs were computed by gene counting, and the chi-squared test was used to calculate the percentages of alleles/genotypes. The SNPs of *TCF7L2* rs7903146 and *MC4R* rs571312 were used to create the GRS. A value varying from zero to two was given to each SNP, indicating the number of metabolic disease-associated risk alleles. The GRS was determined via the addition of the number of risk alleles through each SNP. The median value (1 risk allele) was used to classify the participants into two groups: Those with <1 risk allele and ≥1 risk allele. The association analysis between the GRS and categorical and continuous variables was performed using logistic regression and general linear models, respectively. Logistic and linear regression analyses were performed to examine the interaction between lifestyle factors and SNPs. The models were adjusted for age, gender, obesity status, energy intake, and months of measurement, wherever appropriate. The variable ‘month of measurement’ was created based on the months (June–November) in which the participants were enrolled in the study. The participants included in June, July, and August were coded as ‘Summer’ (*n* = 192 for this group) while the participants included in September, October, and November were coded as ‘Autumn’ (*n* = 204 for this group). *p* value < 0.05 was considered to be statistically significant. The dietary factors and metabolic traits were assessed according to the vitamin D status classified by the IOM recommendation [46]. A power calculation was not conducted given that there are no available effect sizes from studies focusing on metabolic GRS and vitamin D levels in the Turkish population.

## 3. Results

### 3.1. Characteristics of the Study Participants

The mean of serum 25(OH)D concentration was 24.6 ± 1.66 ng/mL in the study population, and the prevalence of VDD was 25% (Table 1). The general characteristics of the study participants including anthropometric measurements, biochemical parameters, dietary intake, and physical activity level are given in Table 1 stratified based on serum vitamin D levels (deficient/insufficient < 20 ng/mL and optimal ≥ 20 ng/mL). No significant difference in clinical, anthropometric, and biochemical parameters was obtained between the groups (*p* > 0.05, for each).

### 3.2. Association of Vitamin D Status with Metabolic Traits

After adjusting for potential confounders, the serum 25(OH)D concentration was significantly associated with the fasting insulin (*p* = 0.011) and HOMA-IR (*p* = 0.010) (Figure S1) and, none of the other phenotypic associations were statistically significant (Table S2).

### 3.3. Genetic Association of Metabolic-GRS with Metabolic Traits and Serum 25(OH)D Concentrations

**Figure 1:**
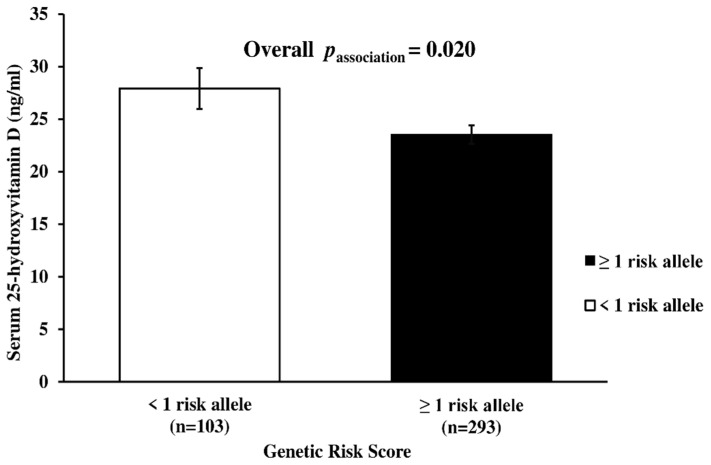
Association between the serum 25-Hydroxy-Vitamin D level and metabolic-GRS. Individuals having 1 or more risk allele had lower serum 25(OH)D concentrations compared to participants with <1 risk allele. The mean and standard deviation for the serum 25(OH)D level was 27.9 ± 1.96 ng/mL in participants with <1 risk allele, while it was 23.5 ± 0.89 ng/mL in participants with ≥1 risk allele. *P* value was calculated using linear regression analysis after adjusting for age, gender, obesity status, and months of measurement.

Metabolic-GRS was significantly associated with the serum 25(OH)D concentration (*p* = 0.020), where participants carrying ≥1 risk allele had lower serum 25(OH)D levels (23.5 ± 0.89 ng/mL) compared to those carrying <1 risk allele (27.9 ± 1.96 ng/mL) (Figure 1). None of the other characteristics differed significantly between the two GRS groups (<1 risk allele vs. ≥1 risk allele) (*p* > 0.05, for all associations) (Table S3).

### 3.4. Interaction between Metabolic-GRS and Serum 25(OH)D Concentration on Clinical and Biochemical Outcomes

There was no significant interaction between metabolic-GRS and vitamin D concentrations on metabolic traits (*p* > 0.05) (Table S4).

### 3.5. Interaction between Metabolic-GRS and Dietary Intake on Serum Vitamin D Concentration

**Figure 2:**
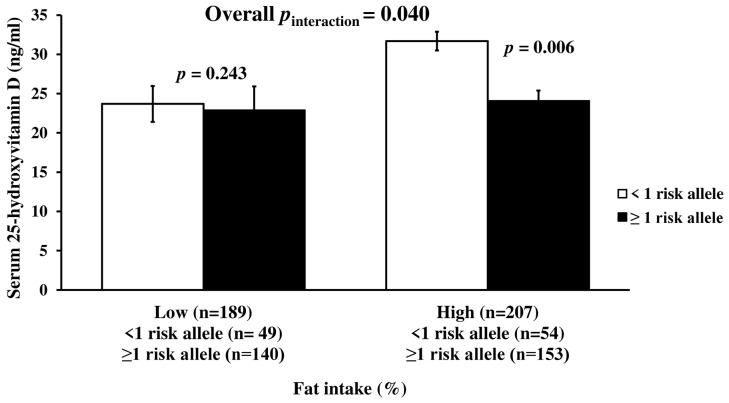
Interaction between metabolic-GRS and fat intake (%) on serum 25(OH)D concentration. There was a significant interaction of the GRS with dietary fat intake on serum 25-hydroxyvitamin D level. Among those with ≥1 risk alleles, individuals with a high of fat intake had a lower serum 25-hydroxyvitamin D level (*p* = 0.006). Vitamin D level was 23.1 ± 1.06 ng/mL among those with low fat intake: For individuals without risk allele: 23.7 ± 2.29; for individuals with risk allele: 22.9 ± 1.19 ng/mL. It was 26.1 ± 1.26 ng/mL among those with a high fat intake (for <1 risk allele: 31.7 ± 3.03; for ≥1 risk alleles: 24.1 ± 1.30 ng/mL). The median value of dietary fat intake was 38%. The mean intake of low-fat intake was 31.6 ± 4.61% (for individuals without risk allele: 31.7 ± 4.89%; for individuals having ≥1 risk alleles: 31.6 ± 4.52%). The mean intake of high fat intake was 44.4 ± 5.32% (for <1 risk allele: 45.1 ± 5.46%; for ≥1 risk alleles: 44.1 ± 5.26%). *p* values were derived from linear regression analysis and adjusted for age, gender, obesity status, and months of measurement.

There was a significant interaction between metabolic-GRS and dietary energy from fat intake on serum 25(OH)D concentrations after adjusting for age, gender, and obesity status, and months of measurement (*p* = 0.040, Figure 2). Participants in the highest tertile of fat intake (122.3 ± 52.51 g/d) and carrying ≥1 risk allele had significantly lower serum 25(OH)D concentrations compared to the participants having the highest tertile of fat intake and carrying <1 risk allele (*p* = 0.006) (Figure 2). No significant interactions between metabolic-GRS and dietary intakes of other macronutrients on serum 25(OH)D were obtained (*p* > 0.05, for each) (Table S5).

## 4. Discussion

To date, our study is the first to use a nutrigenetic approach to investigate the interaction between metabolic-GRS and dietary intakes on serum 25(OH)D levels in a Turkish population. This study proposed a novel interaction between metabolic-GRS and dietary fat intake on serum 25(OH)D concentrations by demonstrating that participants with high metabolic-GRS and higher dietary fat intake had significantly lower serum 25(OH)D levels compared to the participants with high metabolic-GRS but lower dietary fat intake. Given the high prevalence of VDD in Turkey [8,9], these results might have public health significance in preventing VDD in those with high metabolic genetic risk. Therefore, following the current dietary fat intake recommendations (<35%) [52,53] might be important to maintain the optimal vitamin D status, especially in individuals who have a genetic risk of VDD.

Studies that examined the link between metabolic disease associated gene variants and vitamin D status are limited and the findings have been conflicting [25,35]. A recent study conducted in 545 Asian Indians showed no significant association between metabolic-GRS and serum 25(OH)D concentration [25]. On the other hand, Alathari et al. [35] found that Southeast Asian women carrying < 4 metabolic risk alleles had higher serum 25(OH)D concentration compared to the individuals carrying four or more risk alleles. Similarly, our study has shown that individuals having ≥1 metabolic risk allele had lower serum 25(OH)D concentrations than the individuals not having any risk allele. Despite the limited evidence on the link between metabolic disease-associated gene variants and vitamin D levels, many genetic association studies investigated the associations of vitamin D-related SNPs that can modify the activation, catabolism, and transport of vitamin D, with metabolic traits. However, the findings of these studies were also inconsistent [22,54,55,56]. For instance, a couple of studies conducted in European populations showed no association between the gene variants of the vitamin D binding protein/group-specific component (DBP/GC) and the risk of diabetes [54,55], while significant associations have been demonstrated in Asian populations [56]. The discrepancies in the findings of different studies could be explained by the diversity in the number of SNPs, ethnicity, culture, and socioeconomic status.

The present study examined whether the genetic risk of metabolic diseases has been affected by VDD and found no significant interaction between metabolic-GRS and the serum 25(OH)D level on metabolic traits. Similarly, a study that examined the interactions between the vitamin D receptor SNPs and serum vitamin D level on metabolic disease related traits in 5160 Europeans failed to show any evidence of vitamin D-related gene variations modifying the interaction between 25(OH)D concentrations and metabolic traits [57]. Other studies also confirmed the lack of any associations between genetically instrumented serum 25(OH)D concentrations and metabolic traits, such as BMI [35,58,59], waist circumference [35,58,59,60], glycated hemoglobin [35,61], fasting insulin [35,61], and glucose levels [35,61,62].

The World Health Organization Noncommunicable Diseases Progress Monitor (2017) declared that Non-Communicable Diseases (NCDs) have been responsible for 88% of deaths in the Turkish population [63]. Targeting modifiable risk factors for NCDs including the dietary modifications for obesity could prevent mortality [36,64,65]. The present study found that dietary fat intake and metabolic-GRS had an interaction on vitamin D concentrations, and the level of serum 25(OH)D was lower in those carrying risk allele and consuming a high amount of dietary fat. The high amount (≥38% = 122.3 ± 52.51 g/d) was defined according to the median of total dietary fat intake in the study population. This cut off value also meets the high dietary fat intake as defined by the recommendations of WHO (15–30%), IOM (20–35%), and Turkish Dietary Guidelines (20–35%) [52,53,66]. Vitamin D is a fat-soluble vitamin and absorbed with dietary fat by passive diffusion therefore, dietary fat can have a potential to modify the interaction between the genetic risk of metabolic disease and vitamin D status [67]. Similar to current findings, it was shown that high fat diet-induced obesity resulted in lower serum 25(OH)D levels in an animal study [68]. To date, there have been only two studies that have examined the metabolic-GRS- diet interactions on serum 25(OH)D concentrations [25,35]. The first study examined whether any dietary factor could modify the relationship between the serum 25(OH)D concentration and metabolic traits in 545 Asian Indians. In discordance with the findings of our study, they showed that individuals with low GRS (GRS ≤ 1) and lower dietary carbohydrate intake (≤62%) had higher serum 25(OH)D concentrations [25]. Furthermore, the study generated the GRS using five SNPs from three genes (*FTO*, *TCF7L2*, and *MC4R*), and the energy from carbohydrate, protein, and fat was 64%, 11%, and 23%, respectively. The second study tested a similar hypothesis in Southeast Asian Minangkabau women using two GRSs constructed based on 15 SNPs from vitamin D and metabolic disease-associated genes, respectively, and showed no significant interaction between metabolic-GRS and dietary intake on one’s vitamin D status [35]. Some of the reasons for the discrepancy in the findings across the studies might be the number of SNPs that were used in the GRS, ethnicity, and the diversity in the dietary macronutrient intake patterns. Given these ethnic-specific findings, meeting the current dietary recommendations for macronutrient intake might be more essential in individuals with a known genetic risk to help maintain a healthy vitamin D status [52,53,66].

Several hypotheses have been proposed to define the potential mechanisms of the associations between metabolic diseases including obesity and one’s vitamin D status [69,70,71,72,73,74,75,76,77,78]. These include the volumetric dilution of serum vitamin D levels [68,73,74], adipocyte hypertrophy contributing to overexpression of proinflammatory cytokines [77,79], modifications of vitamin D-related enzymes [75,76] affected by high fat diet-induced obesity, and lower endogenous vitamin D synthesis in the skin as a consequence of less outdoor activity [75,80], less physical activity [71], and less exposure to sunlight in obese individuals [73,74,75,76,77,78,79,80]. In addition, the bi-directional Mendelian randomisation analysis conducted in 42,024 Europeans showed a relationship between vitamin D status and obesity, suggesting that higher BMI leads to lower vitamin D levels where a 4.2% decrease in serum 25(OH)D concentrations was observed for every 10% increase in BMI [22]. The Framingham Study also showed that the prevalence of VDD was higher among individuals with a higher BMI [81]. Furthermore, a lifestyle intervention study conducted in obese individuals demonstrated that serum 25(OH)D concentrations were significantly increased as a consequence of weight loss [82]. Despite these findings, some studies failed to show any association between vitamin D status and metabolic traits [83,84,85]. For instance, Larsen et al. [83] showed no or marginal associations between the serum 25(OH)D level and biomarkers of adiposity in 10,898 individuals comprising Danish, British, and Finnish participants. Similarly, independent of the genetic associations, our study also has not shown either any difference in metabolic traits by vitamin D status, or any association between obesity-related traits and the serum 25(OH)D level. The inconsistencies among the studies might depend on the potential predisposition to bias and confounding factors (e.g., the time and amount of sunlight exposure, physical activity level, more clothing, skin colour, and ethnicity) in observational study designs conducted in different populations. Furthermore, the differences in the categorisation of vitamin D status and the measures of obesity including BMI, body weight, and waist circumference might be the other reason for the inconsistency [86]. Genetic studies can provide more consistent findings in the exploration of the association between vitamin D status and metabolic traits, because the bias and confounding factors can be partly eliminated with this approach [26,87].

The main strengths of this study were the use of several biochemical markers related to metabolic traits and a well-characterised study cohort. In addition, the construction and use of a GRS method rather than a single SNP approach enhances the statistical power and presents an efficient perspective for metabolic outcomes [88,89,90]. However, there are some limitations that need to be acknowledged. Firstly, the study did not measure exposure to sunlight, and the data collection period only covered summer and autumn seasons. For overcoming this limitation, the months of measurement was adjusted as a confounding factor in all the analyses. Secondly, the small sample size might be considered as a further limitation of the study however, our study has been able to confirm previously reported associations and identify gene-diet interactions. Thirdly, dietary intake was assessed using a 24-h dietary recall method, which is prone to self-reporting bias however, this method is used commonly in nutrigenetic studies and the method could be applied to diverse groups with a wide range of eating habits. Fourthly, we could not examine the causative effects due to limitations of the cross-sectional study design. Lastly, although analysis undertaken was adjusted for potential confounders, we cannot rule out the impact of residual confounders caused by unknown variables.

## 5. Conclusions

In summary, our study provided evidence for a novel interaction between metabolic-GRS and dietary fat intake on serum vitamin D concentrations, suggesting that following current dietary fat intake recommendations (<35%) might be effective to prevent any consequences of the genetic risk of VDD. However, further larger studies are needed to endorse this interaction before generalising the findings to the Turkish population and implementing any personalised dietary recommendations for the maintenance of one’s optimal vitamin D status.

## Figures and Tables

**Table 1:** Basic characteristics of the study participants according to serum vitamin D levels.

	Serum 25(OH)D Concentration *		
	Deficient/Insufficient (*n* = 182)	Optimal (*n* = 214)	*p* Value
Anthropometric measurements			
Body mass index (kg/m^2^)	25.7 ± 4.21	25.8 ± 4.11	0.271 ^a^
Waist circumference (cm)	87.0 ± 10.79	88.8 ± 12.04	0.938 ^a^
Hip circumference (cm)	101.7 ± 8.27	101.8 ± 7.41	0.127 ^a^
Waist-to-hip ratio	0.86 ± 0.09	0.87 ± 0.08	0.404 ^a^
Fat mass index	6.84 ± 2.96	6.94 ± 2.85	0.559 ^a^
Body fat mass (%)	25.7 ± 7.90	26.0 ± 7.29	0.890 ^a^
Body fat mass (kg)	19.1 ± 7.55	19.6 ± 7.48	0.556 ^a^
Visceral fat percentage	5.59 ± 3.15	5.89 ± 3.25	0.628 ^a^
Biochemical parameters			
Glucose (mg/dL)	88.1 ± 8.21	87.5 ± 8.48	0.305 ^a^
Insulin (µIU/mL)	8.1 ± 0.39	7.3 ± 0.29	0.055 ^a^
Postprandial glucose (mg/dL)	84.9 ± 17.21	84.7 ± 15.72	0.408 ^a^
Postprandial insulin (µIU/mL)	29.3 ± 2.69	24.9 ± 1.95	0.091 ^a^
Very low density lipoprotein (VLDL) cholesterol (mg/dL)	24.1 ± 15.25	23.1 ± 13.76	0.453 ^a^
Total cholesterol (mg/dL)	190.2 ± 40.12	188.0 ± 37.12	0.977 ^a^
High density lipoprotein (HDL) cholesterol (mg/dL)	48.6 ± 11.55	48.8 ± 11.57	0.440 ^a^
Low density lipoprotein (LDL) cholesterol (mg/dL)	123.9 ± 31.20	122.2 ± 28.72	0.913 ^a^
Triglyceride (mg/dL)	120.7 ± 76.35	115.7 ± 68.74	0.440 ^a^
Adiponectin (ng/mL)	10480.1 ± 6217.49	10626 ± 6692.54	0.556 ^a^
Insulin resistance (HOMA-IR)	1.8 ± 0.09	1.6 ± 0.07	0.058 ^a^
Dietary intake			
Total energy (kcal)	2429.3 ± 1093.98	2368.0 ± 992.98	0.675 ^a^
Carbohydrate (%)	46.7 ± 8.90	45.3 ± 9.73	0.073 ^a^
Protein (%)	15.5 ± 3.68	15.7 ± 4.83	0.207 ^a^
Fat (%)	37.5 ± 7.66	38.9 ± 8.41	0.098 ^a^
Total fibre (g)	23.9 ± 10.95	23.7 ± 11.31	0.382 ^a^
Physical activity level, *n* (%)			
Sedentary	68 (37.4)	84 (39.3)	0.306 ^b^
Moderate	90 (49.5)	112 (52.3)	
Vigorous	24 (13.1)	18 (8.4)	

Data are represented as means ± SD for anthropometric measurements, biochemical parameters, and dietary intake; and as number (percentage) for physical activity level. ^a^ Independent sample t test, ^b^ Pearson chi-square test. * Cut-off point for serum vitamin D level was based on the recommendation of the Institute of Medicine.

## Data Availability

The data supporting reported results in the current study are available from the corresponding author on reasonable request.

## Supplementary Materials

The following supporting information can be downloaded at: https://www.mdpi.com/article/10.3390/nu14020382/s1, Figure S1: Association of 25(OH)D concentrations with metabolic traits, Table S1. Genotype frequencies of *TCF7L2* and *MC4R* SNPs, Table S2. Association between the serum 25(OH)D concentration and metabolic traits, Table S3. Metabolic-GRS and baseline characteristics of the study participants, Table S4. The interaction between metabolic-GRS and serum 25(OH) D on metabolic traits, Table S5. The interaction between metabolic-GRS and macronutrient intake on the serum 25(OH)D level.
