# Supplementary material for: Interaction between Dietary Fat Intake and Metabolic Genetic Risk Score on 25-Hydroxyvitamin D Concentrations in a Turkish Adult Population
Source: Nutrients. 2022 Jan 17;14(2):382. doi: 10.3390/nu14020382 (PMC8778439; doi:10.3390/nu14020382)
Supplement: Supplementary file 1 [file nutrients-14-00382-s001.zip › nutrients-1436498-supplementary.pdf]

Table S1. Genotype frequencies of *TCF7L2* and *MC4R* SNPs (n=396)

***TCF7L2* rs7903146**	
**Dominant model, n (%)**	
CC	180 (45.5)
CT+TT	216 (54.5)
MAF	0.34
HWE	0.101
***MC4R* rs571312**	
**Dominant model, n (%)**	
CC	196 (49.5)
CA +AA	200 (50.5)
MAF	0.31
HWE	0.176

MAF: Minor Allele Frequency, HWE: Hardy-Weinberg Equilibrium

Table S2. Association between serum 25(OH)D concentration and metabolic traits

	β	SE	*p**
**Body mass index (kg/m^2)**	-0.012	0.008	0.135
**Waist circumference (cm)**	-0.002	0.003	0.938
**Hip circumference (cm)**	-0.019	0.018	0.312
**Waist-to-hip ratio**	0.002	0.000	0.908
**Waist-to height ratio**	0.006	0.000	0.677
**Fat mass index**	-0.008	0.006	0.159
**Body fat percentage (%)**	-0.022	-0.013	0.097
**Body fat (kg)**	-0.016	0.016	0.330
**Visceral fat percentage (%)**	-0.005	0.006	0.396
**Fasting glucose (mg/dl)**	-0.040	-0.024	0.095
**Fasting insulin (μIU/ml)**	-0.120	0.014	**0.011****
**Postprandial glucose (mg/dl)**	-0.048	0.046	0.296
**Postprandial insulin (μIU/ml)**	-0.161	0.194	0.059
**HOMA-IR**	-0.122	0.003	**0.010****
**VLDL cholesterol (mg/dl)**	-0.028	0.004	0.486
**Total cholesterol (mg/dl)**	-0.071	0.113	0.532
**HDL cholesterol (mg/dl)**	0.010	0.029	0.739
**LDL cholesterol (mg/dl)**	-0.050	0.088	0.565
**Triglycerides (mg/dl)**	-0.142	0.201	0.479
**Adiponectin (ng/ml)**	-2.259	0.006	0.898

**p* values were obtained from linear regression analysis and adjusted for age, gender, obesity status, and month of measurement.
***p*<0.05

Table S3. Metabolic-GRS and baseline characteristics of the study participants

	Metabolic-GRS		
	**<1 risk allele (n=103)**	**≥1 risk allele (n=293)**	***p* value**
**Anthropometric measurements**			
Body mass index (kg/m^2)	25.6±4.08	25.8±4.18	0.548[a]
Waist circumference (cm)	87.5±11.11	88.0±11.66	0.697[a]
Hip circumference (cm)	101.4±7.58	101.9±7.89	0.523[a]
Waist-to-hip ratio	0.86±0.07	0.87±0.09	0.825[a]
Waist-to-height ratio	0.52±0.06	0.52±0.07	0.858[a]
Fat-to- muscle ratio	0.38±0.15	0.39±0.17	0.521[a]
Fat mass index	6.79±2.68	6.92±2.97	0.558[a]
Body adiposity index	22.2±4.79	22.1±5.38	0.658[a]
Body fat mass (%)	25.8±7.45	25.9±7.62	0.726[a]
Body fat mass (kg)	18.9±7.28	19.5±7.59	0.389[a]
Bone mineral density (kg)	2.7±0.55	2.8±0.51	0.206[a]
Visceral fat percentage	5.6±2.89	5.9±3.31	0.368[a]
**Biochemical parameters**			
Glucose (mg/dl)	86.5±7.40	88.2±8.62	0.076[a]
Insulin (µIU/ml)	7.9±5.09	7.6±4.67	0.526[a]
Postprandial glucose (mg/dl)	84.8±13.51	84.8±17.33	0.894[a]
Postprandial insulin (µIU/ml)	26.7±29.19	27.0±33.46	0.978[a]
VLDL cholesterol (mg/dl)	21.6±12.11	24.3±15.14	0.086[a]
Total cholesterol (mg/dl)	189.0±42.58	189.0±37.02	0.902[a]
HDL cholesterol (mg/dl)	49.9±11.13	48.3±11.68	0.151[a]
LDL cholesterol (mg/dl)	122.9±33.41	123.0±28.57	0.926[a]
Triglyceride (mg/dl)	107.9±60.56	121.5±75.76	0.091[a]
Adiponectin (ng/ml)	10322.5±5920.02	10642.1±6661.27	0.595[a]
HOMA-IR	1.7±1.11	1.7±1.20	0.890[a]
Serum 25 (OH) D (ng/ml)	27.9±1.96	23.5±0.89	**0.020[b*]**
**Dietary intake**			
Total energy (kcal)	2345.9±894.81	2410.7±1087.13	0.655[a]
Carbohydrate (%)	45.4±9.24	46.1±9.43	0.529[a]
Protein (%)	15.7±4.87	15.6±4.14	0.887[a]
Fat (%)	38.7±8.48	38.1±7.96	0.520[a]
Total Fiber (g)	22.5±10.02	24.2±11.48	0.196[c]
**Physical activity level, n (%)**			
Sedentary	39 (37.9)	113 (38.6)	0.923[d]
Moderate	52 (50.5)	150 (51.2)	
Vigorous	12 (11.7)	30 (10.2)	

GRS: Genetic risk score
*p<0.05
[a,b,c] Linear regression analysis; [a]Adjusted for age, gender, obesity status, [b] Adjusted for age, gender, obesity status and month of measurement [c]Adjusted for age, gender, obesity status, energy intake, [d]Pearson chi-square test

Table S4. The interaction between metabolic-GRS and serum 25(OH) D on metabolic traits*

**Metabolic disease outcomes**	$p_{interaction}$
Fasting plasma glucose	0.749
Fasting plasma insulin	0.169
Plasma postprandial glucose	0.850
Plasma postprandial insulin	0.147
HOMA-IR	0.313
Serum VLDL cholesterol	0.808
Serum total cholesterol	0.477
Serum HDL cholesterol	0.254
Serum LDL cholesterol	0.588
Serum triglycerides	0.470
Plasma adiponectin	0.593
Systolic blood pressure	0.245
Diastolic blood pressure	0.918

GRS: Genetic risk score

**p* values were obtained from linear regression analysis, and adjusted for age, gender, obesity status, and month of measurement.

Table S5. The interaction between metabolic-GRS and macronutrient intake on serum 25(OH) D level*

$p_{interaction}$ for energy from protein intake (%) = 0.968
$p_{interaction}$ for energy from carbohydrate intake (%) = 0.213
$p_{interaction}$ for energy from fat intake (%) = **0.040****

GRS: Genetic risk score

*p values were obtained from linear regression analysis, and adjusted for age, gender, obesity status, and month of measurement.

**p<0.05

S1A.

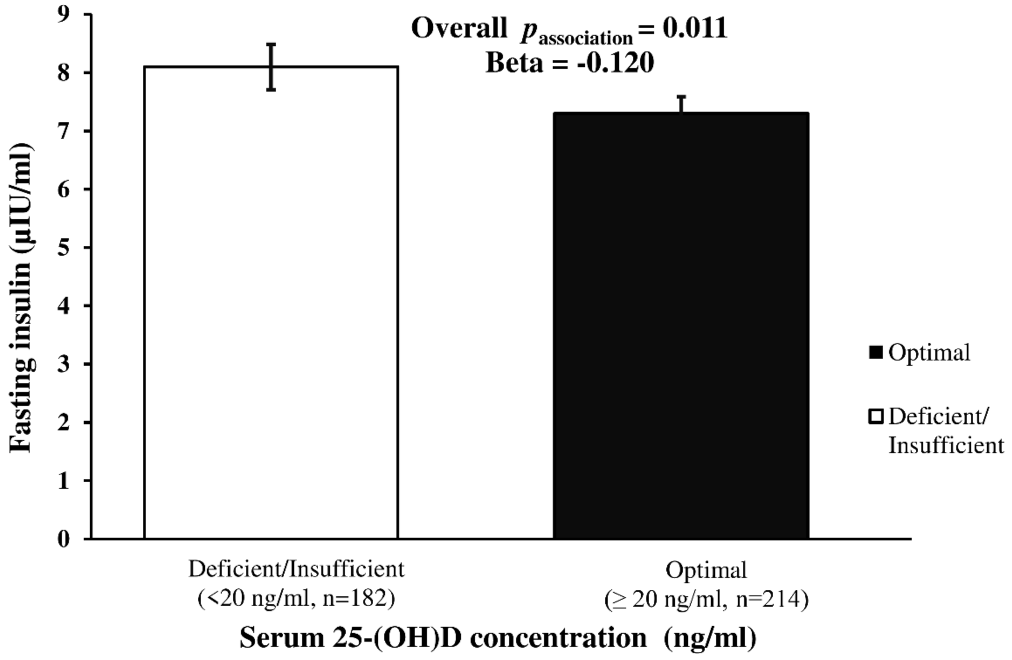

S1B.

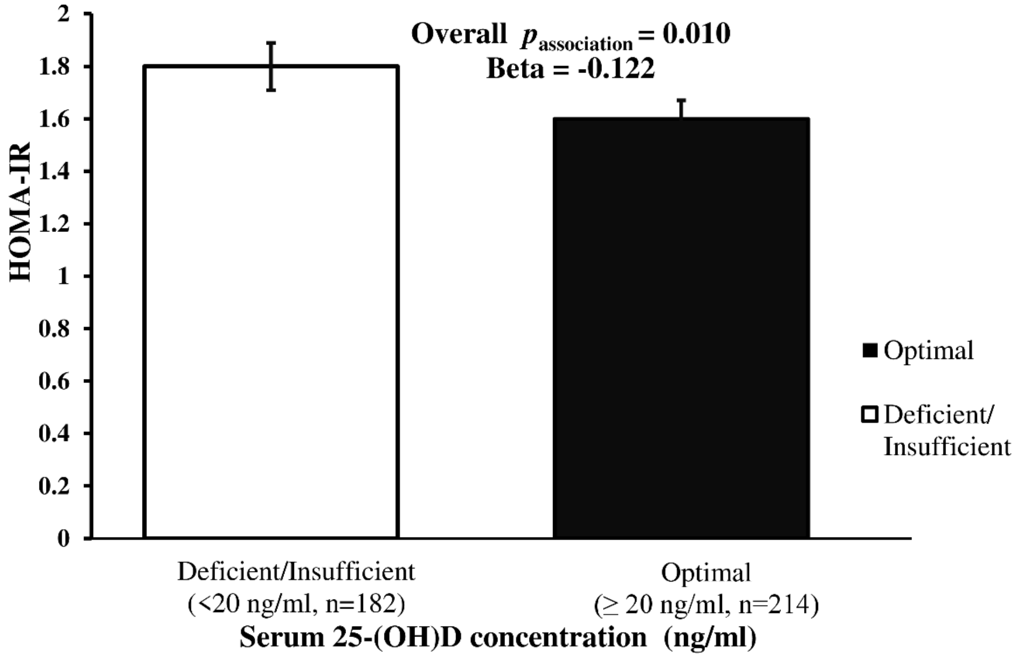

Figure S1. Association of 25(OH)D concentrations with metabolic traits. There was an inverse association between serum 25(OH)D concentration and fasting insulin concentration (p=0.011) and HOMA-IR (p=0.010). A) Individuals with optimal serum 25 (OH)D concentration had lower plasma fasting insulin level (8.1±0.39 vs. 7.3±0.29 µIU/ml).

B) Individuals with optimal serum 25 (OH)D concentration had lower HOMA-IR value (1.8±0.09 vs. 1.6±0.07). p values were obtained from linear regression analysis and adjusted for age, gender, seasonal variation, and obesity status.
